# Supplementary material for: A HaloTag-TEV genetic cassette for mechanical phenotyping of proteins from tissues
Source: Nat Commun. 2020 Apr 28;11:2060. doi: 10.1038/s41467-020-15465-9 (PMC7189229; doi:10.1038/s41467-020-15465-9)
Supplement: Supplementary file 3 — Description of Additional Supplementary Files [file 41467_2020_15465_MOESM3_ESM.pdf]

Title: Supplementary Movie 1.
Description: 3D reconstruction of HaloTag-TEV clarified muscle fibers. Following specific labeling of gastrocnemius muscle with Oregon Green Halo ligand, fixation and clarification, a 250-μm-deep Z-stack was obtained using multiphoton microscopy (127 images). Individual images were intensity corrected and the software Imaris was used to produce the 3D animation.
