# Supplementary material for: A HaloTag-TEV genetic cassette for mechanical phenotyping of proteins from tissues
Source: Nat Commun. 2020 Apr 28;11:2060. doi: 10.1038/s41467-020-15465-9 (PMC7189229; doi:10.1038/s41467-020-15465-9)
Supplement: Supplementary file 4 — Reporting Summary [file 41467_2020_15465_MOESM4_ESM.pdf]

nature research

Corresponding author(s): Wolfgang A. Linke, Jorge Alegre-Cebollada

Last updated by author(s): 14/february/20

Reporting Summary

Nature Research wishes to improve the reproducibility of the work that we publish. This form provides structure for consistency and transparency in reporting. For further information on Nature Research policies, seeAuthors & Referees and theEditorial Policy Checklist .

Statistics

For all statistical analyses, confirm that the following items are present in the figure legend, table legend, main text, or Methods section.

n/a	Confirmed	
☐	☒	The exact sample size (*n*) for each experimental group/condition, given as a discrete number and unit of measurement
☐	☒	A statement on whether measurements were taken from distinct samples or whether the same sample was measured repeatedly
☐	☒	The statistical test(s) used AND whether they are one- or two-sided<br>*Only common tests should be described solely by name; describe more complex techniques in the Methods section.*
☒	☐	A description of all covariates tested
☒	☐	A description of any assumptions or corrections, such as tests of normality and adjustment for multiple comparisons
☐	☒	A full description of the statistical parameters including central tendency (e.g. means) or other basic estimates (e.g. regression coefficient) AND variation (e.g. standard deviation) or associated estimates of uncertainty (e.g. confidence intervals)
☐	☒	For null hypothesis testing, the test statistic (e.g. *F*, *t*, *r*) with confidence intervals, effect sizes, degrees of freedom and *P* value noted<br>*Give P values as exact values whenever suitable.*
☒	☐	For Bayesian analysis, information on the choice of priors and Markov chain Monte Carlo settings
☒	☐	For hierarchical and complex designs, identification of the appropriate level for tests and full reporting of outcomes
☒	☐	Estimates of effect sizes (e.g. Cohen's *d*, Pearson's *r*), indicating how they were calculated

Our web collection on statistics for biologists contains articles on many of the points above.

Software and code

Policy information about availability of computer code

Data collection	Muscle mechanics experiments were acquired in Asci Format unsing a Scientific Instruments (Heidelberg, Germany) workstation and custom-made software. Magnetic tweezers run using custom-written software in igor. Echocardiography measurements were taken in a commercial Veevo 2100 workstation.
Data analysis	Fluorescence images were analyzed using Leica suited software and Image J. Magnetic tweezers data wre analyzed custom-written software in Igor 6. Muscle mechanics data were analyzed in Origin. Echocardiography data was analyzed using the Vevo 2100 Workstation software.

For manuscripts utilizing custom algorithms or software that are central to the research but not yet described in published literature, software must be made available to editors/reviewers. We strongly encourage code deposition in a community repository (e.g. GitHub). See the Nature Research guidelines for submitting code & software for further information.

Data

Policy information about availability of data

All manuscripts must include a data availability statement. This statement should provide the following information, where applicable:

- Accession codes, unique identifiers, or web links for publicly available datasets
- A list of figures that have associated raw data
- A description of any restrictions on data availability

Data and materials are available on reasonable request to the corresponding authors.

Field-specific reporting

Please select the one below that is the best fit for your research. If you are not sure, read the appropriate sections before making your selection.

- [x] Life sciences
- [ ] Behavioural & social sciences
- [ ] Ecological, evolutionary & environmental sciences

For a reference copy of the document with all sections, see nature.com/documents/nr-reporting-summary-flat.pdf

Life sciences study design

All studies must disclose on these points even when the disclosure is negative.

Sample size	No specific sample size calculation was undertaken before experiments. We chose sample sizes according to standard practices in the fields of single-molecule biophysics and muscle physiology.
Data exclusions	On the advise of reviewer #3, we excluded sarcomere length data that was not compatible with published evidence (probably due to residual contraction of the samples measured).
Replication	The results from TEV digestion experiments were variable. As discussed in the paper, we verified full digestion of HaloTag-TEV-titin by SDS-PAGE to estimate titin contribution to stiffness. The experiments to assess the general health status of the mice were done once since we consider replication would not add relevant information given the results. In general, all the microscopy experiments shown in the paper are representative of several replicates. Number of molecules analyzed in single-molecule data are reported in the paper.
Randomization	No comparisons between treatment groups are done - randomization does not apply
Blinding	Blinding was used for the collection and analysis of echocardiography data and serum markers. For the remaining comparisons between wild-type and knock-in animals, we consider blinding was not relevant to our study since comparison between groups was not the key point

Reporting for specific materials, systems and methods

We require information from authors about some types of materials, experimental systems and methods used in many studies. Here, indicate whether each material, system or method listed is relevant to your study. If you are not sure if a list item applies to your research, read the appropriate section before selecting a response.

Materials & experimental systems

n/a	Involved in the study	
[ ]	[x]	Antibodies
[x]	[ ]	Eukaryotic cell lines
[x]	[ ]	Palaeontology
[ ]	[x]	Animals and other organisms
[x]	[ ]	Human research participants
[x]	[ ]	Clinical data

Methods

n/a	Involved in the study	
[x]	[ ]	ChIP-seq
[x]	[ ]	Flow cytometry
[x]	[ ]	MRI-based neuroimaging

Antibodies

Antibodies used	T12, MIR (anti titin)
Validation	Furst et al, J Cell Biol. 1988 May;106(5):1563-72; Gautel et al, Neurology. 1993 Aug;43(8):1581-5., Linke et al, J Mol Biol 1996 Aug 9;261(1):62-71

Animals and other organisms

Policy information about studies involving animals; ARRIVE guidelines recommended for reporting animal research

Laboratory animals	Mus musculus, age indicated in the paper, both male and female, strain: SW (SWR/J) and C57BL/6
Wild animals	none
Field-collected samples	none
Ethics oversight	Area de Protección Animal de la Comunidad de Madrid (PROEX 042/18)<br>Animal Care and Use Committee of the University Clinic Muenster (A18.016)

Note that full information on the approval of the study protocol must also be provided in the manuscript.
